# Salusin-β in Intermediate Dorsal Motor Nucleus of the Vagus Regulates Sympathetic-Parasympathetic Balance and Blood Pressure

**DOI:** 10.3390/biomedicines9091118

**Published:** 2021-08-31

**Authors:** Lu-Lu Wu, Jin-Hua Bo, Fen Zheng, Feng Zhang, Qi Chen, Yue-Hua Li, Yu-Ming Kang, Guo-Qing Zhu

**Affiliations:** 1 Key Laboratory of Targeted Intervention of Cardiovascular Disease, Collaborative Innovation Center of Translational Medicine for Cardiovascular Disease, and Department of Physiology, Nanjing Medical University, Nanjing 211166, China; luluuu@njmu.edu.cn (L.-L.W.); bojinhua@njglyy.com (J.-H.B.); fenzh@njmu.edu.cn (F.Z.); fengzhang@njmu.edu.cn (F.Z.); 2 Department of Anesthesiology, Nanjing Drum Tower Hospital, Clinical College of Nanjing Medical University, Nanjing 210008, China; 3 Department of Pathophysiology, Nanjing Medical University, Nanjing 211166, China; qichen@njmu.edu.cn (Q.C.); yhli@njmu.edu.cn (Y.-H.L.); 4 Department of Physiology and Pathophysiology, Cardiovascular Research Center, Xi’an Jiaotong University School of Medicine, Xi’an 710061, China; ykang@mail.xjtu.edu.cn

**Keywords:** salusin, dorsal motor nucleus of the vagus, sympathetic activity, blood pressure, reactive oxygen species, paraventricular nucleus, γ-aminobutyric acid

## Abstract

The dorsal motor nucleus of the vagus (DMV) is known to control vagal activity. It is unknown whether the DMV regulates sympathetic activity and whether salusin-β in the DMV contributes to autonomic nervous activity. We investigated the roles of salusin-β in DMV in regulating sympathetic-parasympathetic balance and its underline mechanisms. Microinjections were carried out in the DMV and hypothalamic paraventricular nucleus (PVN) in male adult anesthetized rats. Renal sympathetic nerve activity (RSNA), blood pressure and heart rate were recorded. Immunohistochemistry for salusin-β and reactive oxidative species (ROS) production in the DMV were examined. Salusin-β was expressed in the intermediate DMV (iDMV). Salusin-β in the iDMV not only inhibited RSNA but also enhanced vagal activity and thereby reduced blood pressure and heart rate. The roles of salusin-β in causing vagal activation were mediated by NAD(P)H oxidase-dependent superoxide anion production in the iDMV. The roles of salusin-β in inhibiting RSNA were mediated by not only the NAD(P)H oxidase-originated superoxide anion production in the iDMV but also the γ-aminobutyric acid (GABA)_A_ receptor activation in PVN. Moreover, endogenous salusin-β and ROS production in the iDMV play a tonic role in inhibiting RSNA. These results indicate that salusin-β in the iDMV inhibits sympathetic activity and enhances vagal activity, and thereby reduces blood pressure and heart rate, which are mediated by NAD(P)H oxidase-dependent ROS production in the iDMV. Moreover, GABA_A_ receptor in the PVN mediates the effect of salusin-β on sympathetic inhibition. Endogenous salusin-β and ROS production in the iDMV play a tonic role in inhibiting sympathetic activity.

## 1. Introduction

Autonomic nervous system dysfunction is closely associated with cardiovascular diseases, including hypertension, chronic heart failure and chronic kidney disease [1,2,3]. Inhibition of the enhanced sympathetic activity such as renal denervation has been used as an important strategy for intervention of these diseases [4,5,6]. Many studies are focused on the roles and mechanisms of hypothalamic periventricular nucleus (PVN), rostral ventrolateral medulla (RVLM) and the nucleus of the solitary tract (NTS) in regulating sympathetic activity [7,8,9]. Interventions primarily aim to inhibit excessive sympathetic activation, while vagal activity has been largely neglected. The central control of the sympathetic-parasympathetic balance is not well elucidated.

The dorsal motor nucleus of the vagus (DMV) serves as a key integrative site of parasympathetic neuronal network [10,11]. The axons of cardiac vagal preganglionic neurons are found in the intermediate zone of DMV (iDMV) [12]. Activity of vagal preganglionic neurons in the DMV is responsible for tonic parasympathetic control of ventricular excitability [13]. Microinjection of glycine into the DMV increases blood pressure and heart rate (HR) in rats [14], while glutamate reduces blood pressure and HR [15], suggesting that the activation of the DMV causes depressor and bradycardia responses. However, it is still unknown whether the DMV would regulate sympathetic activity and the balance between the sympathetic and parasympathetic outflow.

Salusins are originally identified in 2003 from full-length cDNAs in human by bioinformatics analyses, including two bioactive peptides of 28 and 20 amino acids named salusin-α and salusin-β [16]. The initial 18 amino acids of human salusin-β have a high homology with the N-terminal sequence of rat salusin [17]. Rat salusin is immunologically similar to human salusin-β, and is widely expressed in brain, peripheral tissues and serum [17,18]. Salusin-β promotes vascular smooth muscle cell (VSMC) proliferation and migration [19,20], and knockdown of salusin-β attenuates cardiovascular remodeling and hypertension in spontaneously hypertensive rats (SHR) [21]. Our previous studies have shown that salusin-β in PVN and RVLM increases sympathetic outflow and blood pressure in SHR, but not in normal rats [22,23]. Microinjection of salusin-β in NTS of SHR reduces sympathetic outflow and blood pressure [24]. We found a lot of salusin-β-positive neurons in the iDMV. However, it is still unknown whether salusin-β in the DMV is involved in the regulation of sympathetic and parasympathetic activity and blood pressure. This study is designed to investigate the role of salusin-β in regulating sympathetic/parasympathetic activity and blood pressure, and its underline mechanisms in rats.

## 2. Materials and Methods

### 2.1. Animals

Male Sprague–Dawley (SD) rats weighing between 300 and 340 g were used in the present study. The experimental protocols were conformed to the recommendations in the Guide for the Care and Use of Laboratory Animals (8th edn, NIH) and approved by the Experimental Animal Care and Use Committee ((IACUC-1811017, Nov, 2018; Nanjing Medical University, China). The rats were kept in an environment under a 12-h cycle of light/dark with controlled temperature and humidity. Standard laboratory chow and tap water were available ad libitum. A total of 7 rats were excluded from the study. Among them, 1 rat failed due to bleeding in surgery, 2 rats in unsustainable recording of sympathetic nerve activity (RSNA), and 4 rats in the inaccurate microinjection sites.

### 2.2. General Procedures

Rat was anaesthetized with intraperitoneal injection of urethane (800 mg/kg) and α-chloralose (40 mg/kg). The rat was kept supine, and the trachea and carotid artery were exposed via a vertical incision in the middle of the neck. Positive pressure ventilation was carried out with room air via endotracheal intubation using a small animal ventilator (Model 683, Harvard Apparatus Inc., Holliston, MA, USA). A PE50 catheter was intubated into right common carotid artery and connected with a pressure transducer (MLT0380, ADInstruments, Bella Vista, NSW, Australia) for blood pressure recording. A left flank incision was made to expose the left kidney and renal nerves for preparing recording of RSNA. The sympathetic nerve activity (RSNA), mean arterial pressure (MAP) and heart rate (HR) were simultaneously recorded with a data acquisition system (8SP, ADInstruments, Bella Vista, NSW, Australia). The rat was allowed to stabilize for more than 30 min before experimental intervention. Finally, the animal was euthanized by rapid intravenous injection of pentobarbital sodium (Sigma, St. Louis, MO, USA) at the dose of 100 mg/kg.

### 2.3. RSNA Recording

A left flank incision was performed, and left renal nerve was isolated. The nerve was cut at the distal end to eliminate the afferent activity of the kidney. The central end of the nerve was put on a pair of platinum electrodes, and immersed in mineral oil at 37 °C. The renal nerve signals were amplified using a differential amplifier (model DP-304, Warner Instruments, Hamden, CT, USA) with a band-pass filtration at 100–3000 Hz. The raw RSNA were integrated at a time constant of 100 ms using LabChart 8 software (ADInstruments, Bella Vista, NSW, Australia). Background noise was obtained after cutting at the central end of the renal nerve. The percentage change of integrated RSNA from the baseline value was calculated after each intervention.

### 2.4. DMV Microinjection

Rat was fixed with a stereotaxic frame (Stoelting, Chicago, IL, USA) in a prone position. The skull was exposed via an incision at the midline of the scalp. Three microinjection sites were selected for representing rostral, intermediate and caudal zones of DMV respectively according to the Paxinos and Watson rat atlas [25]. The coordinates for the rostral zone of DMV (rDMV) were 12.72 mm caudal to the bregma, 0.91 mm lateral to the midline, and 7.82 mm deep from the dorsal surface. The coordinates for the intermediate zone of DMV (iDMV) were 13.56 mm caudal to the bregma, 0.60 mm lateral to the midline, and 7.92 mm deep from the dorsal surface. The coordinates for the caudal zone of DMV (cDMV) were 14.40 mm caudal to the bregma, 0.34 mm lateral to the midline, and 8.08 mm deep from the dorsal surface. Bilateral DMV microinjections were performed via glass micropipettes (tip size 50 μm) connected to a 0.5 μL microsyringe, and completed within 1 min. The volume of microinjection was 50 nL for each side of the DMV. At the end of the experiment, the same volume of Evans Blue was microinjected into the DMV preparing for histological identification. The data for the microinjection sites outside the DMV were excluded.

### 2.5. Heart Rate Variability (HRV) Analysis

HRV is used to quantitatively evaluate the activity of sympathetic and parasympathetic nerves. High-frequency (HF) indicates parasympathetic activity, while Low-frequency (LF) reflects both sympathetic and parasympathetic activities. The LF/HF ratio is mainly used as an effective indicator of sympathetic activity- [26]. Electrocardiograph (ECG) was recorded with subcutaneous electrodes of standard II configuration sing the PowerLab system (4/25T, ADInstruments, Bella Vista, NSW, Australia) for HRV analysis. HRV was measured in the frequency domain. We evaluated the LF power at 0.20 to 0.75 Hz, the HF power at 0.75 to 2.50 Hz, the very low frequency power (VLF) at 0 to 0.20 Hz, and the total power (TP) at 0 to 3.00 Hz. The nLF and the nHF values were used in the present study, which can reflect sympathetic-parasympathetic activity better. The nLF serves as an index of sympathetic activity, and nHF is an index of parasympathetic activity, which are calculated by the following equations. nLF = 100 × LF/(TP − VLF); nHF = 100 × HF/(TP − VLF). The values are expressed as normalized units (nu). The nLF/nHF ratio represents the state of sympathetic-parasympathetic balance [27,28].

### 2.6. In-Situ Detection of Superoxide Anions in DMV

Dihydroethidium (DHE) (Beyotime Biotechnology, Shanghai, China) was used as a specific fluorogenic probe to detect superoxide anions in the DMV as we reported previously [29]. Samples from PBS- and salusin-β-treated rats were processed in parallel. The rats were euthanized with an overdose of pentobarbital as we mentioned above. Brains were rapidly removed, frozen with liquid nitrogen, embedded into tissue OCT-Freeze Medium, and cryostat sectioned (30 μm, coronal) onto chilled microscope slides. The sections were thawed at room temperature, rehydrated with phosphate-buffered saline, and incubated for 5 min in the dark with DHE (1 μmol/L). After washing with phosphate-buffered saline, the DHE fluorescence in sections was detected with a fluorescence microscope (BX51, Olympus, Tokyo, Japan) using an excitation wavelength of 543 nm and a rhodamine emission filter. The detector and laser settings were kept constant among all samples.

### 2.7. Measurement of Superoxide Anion Level and NAD(P)H Oxidase Activity

Coronal sections at a 450-µm-thickness were performed at the iDMV level with a cryostat microtome (Model CM1900, Leica, Wetzlar, Germany). The bilateral iDMV areas were punched out with a 15-gauge needle. The punched tissues in lysis buffer were homogenized and centrifuged. The total protein concentration was measured with the Bradford assay (BCA; Pierce, Santa Cruz, CA, USA). Superoxide anion levels and NAD(P)H oxidase activity were measured with lucigenin-derived chemiluminescence method [30]. For measuring superoxide anion levels, the photon emission was triggered by adding dark-adapted lucigenin (5 μM). For examining the NAD(P)H oxidase activity, the photon emission was initiated by adding both NAD(P)H (100 μM) and dark-adapted lucigenin (5 μM). Light emission was measured with a luminometer (Model 20/20 n, Turner, CA, USA) ten times in 10 min. Background chemiluminescence was measured in the buffer containing lucigenin (5 μM). Mean light unit (MLU)/min/mg protein represented the superoxide anion levels and NAD(P)H oxidase activity in the samples.

### 2.8. Immunohistochemistry

Immunohistochemistry analysis was performed as we previously reported [31,32] Brain sections were respectively performed at the level of iDMV. Immunohistochemistry for salusin-β was made. The paraffin-embedded samples were rehydrated and immersed in 3% hydrogen peroxide for 15 min to quench the endogenous peroxidase. The sections were blocked in 10% goat serum for 1 h at room temperature, and incubated with the salusin-β antibody overnight at 4 °C. The immune positive signals were detected with DAB substrate-chromogen solution for 5 min following the manufacturer’s suggestions. Finally, sections were counterstained with haematoxylin. The salusin-β positive neurons and fibers in the DMV were detected with a light microscopy (DP70, Olympus, Tokyo, Japan).

### 2.9. Vagotomy and Sinoaortic Denervation (SAD)

In examining the effects of vagi and baroreflex on the roles of salusin-β, vagotomy or SAD was carried out in rats, respectively. For the rats with vagotomy, bilateral vagi were isolated in the neck, tied and sectioned. For the rats with SAD, bilateral vagi were kept intact avoiding damage. The carotid bifurcation and the common carotid arteries were stripped of adventitial tissues from 4 mm below the bifurcation to 4 mm above. Similarly, aortic arch was stripped of adventitial tissues. The vessels were painted with 10% phenol solution to destroy any remaining nerve fibers in the sections of arteries. The effectiveness of SAD was determined by the HR response to phenylephrine (20 µg/kg, iv)-induced pressor effect (25–40 mmHg). SAD was confirmed by the HR change that was less than 5 beats/min in response to phenylephrine (Sinopharm Chemical Reagent Co., Ltd., Shanghai, China).

### 2.10. Chemicals and Antibodies

Salusin-β was purchased from Phoenix Pharmaceuticals (Burlingame, CA, USA). Tempol, apocynin and CGP35348 were obtained from Sigma (St. Louis, MO, USA). Bicuculline was obtained from MedChemExpress (Shanghai, China). Salusin-β, tempol and bicuculline were dissolved in PBS. Apocynin and CGP35348 were dissolved in PBS containing 1% DMSO. Vehicle was used as a control. Rabbit anti-salusin-β IgG and rabbit anti-salusin-β serum were obtained from Bachem (Bubendorf, Switzerland). The antibody specificity has been identified by radioimmunoassay, and the anti-salusin-β IgG had no cross reaction with salusin-α.

### 2.11. Statistics

Experiments were performed in a double-blinded and randomized fashion. The RSNA, MAP and HR responses to chemicals were determined by a 1-min average at the chemical-induced maximal responses and at the baseline values before application of chemicals. Data were expressed as mean ± SE. Student’s *t*-test was used for comparing the difference between two groups. One-way or two-way ANOVA followed by Bonferroni’s post hoc analysis were used for multiple comparisons. A *p*-value less than 0.05 was considered statistically significant.

## 3. Results

### 3.1. Effects of Salusin-β in Different Zones of DMV

**Figure 1:**
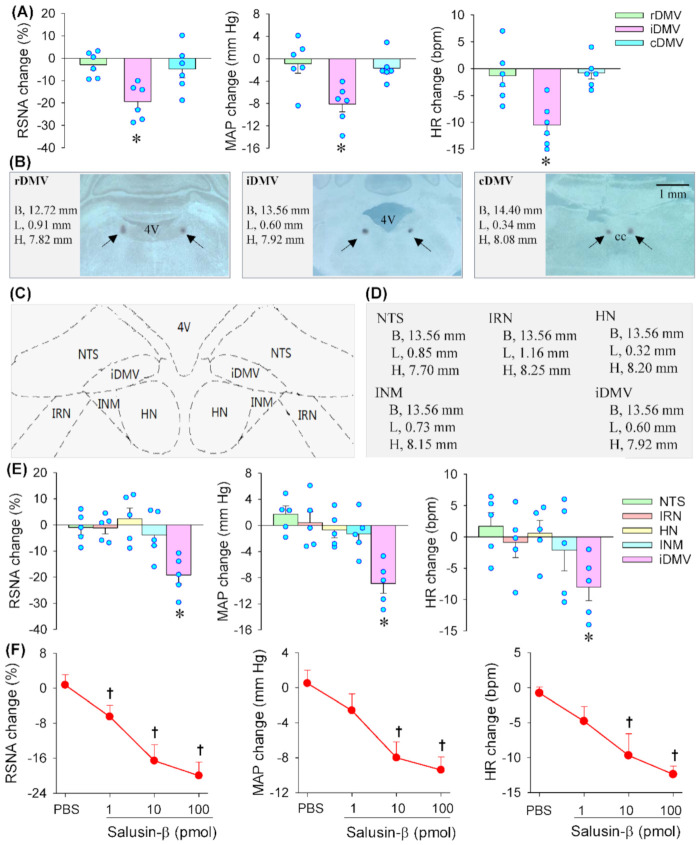
Effects of microinjection of salusin-β in dorsal motor nucleus of the vagus (DMV) on RSNA, MAP and HR. (**A**) Effects of salusin-β (100 pmol) in different zones of DMV including rostral DMV (rDMV), intermediate DMV (iDMV) and caudal DMV (cDMV). (**B**) Representative images of DMV showing microinjection sites at rDMV, iDMV and cDMV. (**C**) Schematic diagram showing the nuclei around the iDMV such as nucleus tractus solitaries (NTS), intermediate reticular nucleus (IRN), hypoglossal nucleus (HN) and intermedius nucleus of medulla (INM). (**D**) The coordinates used for microinjection of salusin-β into the NTS, IRN, HN, INM and iDMV. (**E**) Effects of microinjection of salusin-β (100 pmol) into the NTS, IRN, HN, INM and iDMV. (**F**) Effects of different doses of salusin-β (1, 10, 100 pmol) in the intermediate zone of DMV (iDMV).* *p* < 0.05 vs. the value before microinjection. † *p* < 0.05 vs. PBS. 4V, the fourth ventricle; CC, central canal.

Microinjection of salusin-β (100 pmol) into the iDMV significantly reduced RSNA, MAP and HR. However, microinjection of the same dose of salusin-β into the rDMV or cDMV failed to cause significant changes in RSNA, MAP and HR (Figure 1A). The microinjection sites and coordinates were shown with representative images (Figure 1B). According to the Paxinos and Watson rat atlas [25], there are several nuclei round the iDMV including NTS, intermediate reticular nucleus (IRN), hypoglossal nucleus (HN) and intermedius nucleus of medulla (INM), which were shown in a schematic diagram (Figure 1C). In order to exclude the possibility that the effects of salusin-β in the iDMV were caused by its diffusion to other brain areas, microinjection of salusin-β in the NTS, IRN, HN and INM were respectively carried out. The microinjection sites for these nuclei were determined based on Paxinos and Watson rat atlas [25], and the coordinates used for microinjection in these nuclei were listed (Figure 1D). Microinjection of salusin-β in the NTS, IRN, HN and INM had no significant effects on the RSNA, MAP and HR (Figure 1E). These results confirmed the effects of salusin-β in the iDMV. Therefore, we investigated the effects of salusin-β in iDMV rather than rDMV, cDMV and other nuclei around the iDMV in the following studies.

### 3.2. Dose-Effects and Time-Effects of Salusin-β in iDMV

**Figure 2:**
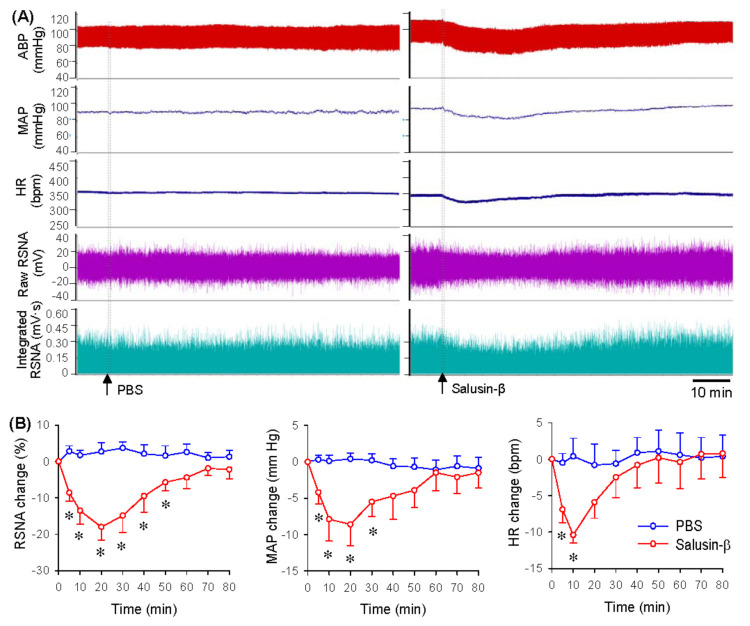
Time-effects of salusin-β in iDMV on RSNA, MAP and HR. (**A**) Representative recordings showing the effects of microinjection of PBS and salusin-β (100 pmol) in iDMV. (**B**) Line graph showing the time-effects of microinjection of PBS and salusin-β (100 pmol) in iDMV. * *p* < 0.05 vs. PBS. *n* = 6 per group.

Microinjection of different doses of salusin-β (0, 1, 10 or 100 pmol) into the iDMV dose-dependently reduced RSNA, MAP and HR (Figure 1F). The salusin-β at the dose of 100 pmol was almost reached its maximal effects. Thus, the dose at 100 pmol was used for investigating the effects of salusin-β in the iDMV in the present study, which was consistent with our previous studies that 100 pmol of salusin-β was used to test the effects of salusin-β in the RVLM and the PVN [22,23]. Representative recordings showing that microinjection of PBS into the iDMV had no significant cardiovascular effects, but salusin-β caused a rapid reduction in RSNA, MAP and HR (Figure 2A). The effects of salusin-β last 30–50 min, reaching its maximal effects at about 10–20 min (Figure 2B).

### 3.3. Roles of Vagi in the Effects of Salusin-β in the iDMV

**Figure 3:**
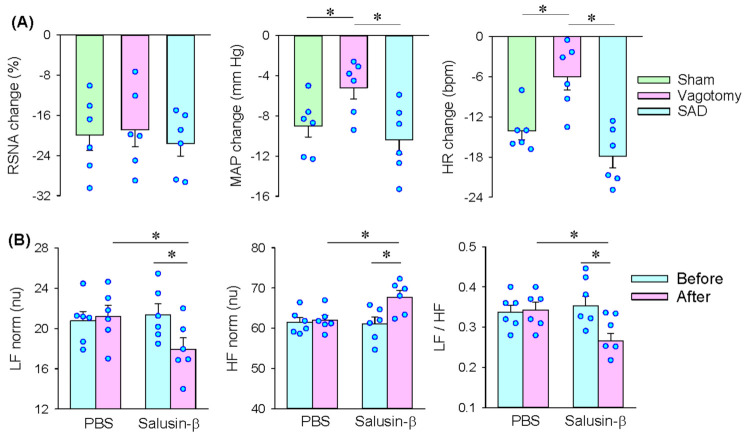
Roles of vagi in the effects of microinjection of salusin-β (100 pmol) in the iDMV. (**A**) Effects of bilateral vagotomy or sinoaortic denervation (SAD) on the roles of microinjection of salusin-β (100 pmol) in iDMV. (**B**) Effects of microinjection of salusin-β (100 pmol) in the iDMV on heart rate variability (HRV). nLF, normalized low frequency; nHF, normalized high frequency. * *p* < 0.05.

Bilateral vagotomy attenuated the roles of salusin-β in the iDMV in reducing MAP and HR but failed to alter the roles of salusin-β in inhibiting the RSNA. SAD had no significant effects on the roles of salusin-β in inhibiting the RSNA, MAP and HR (Figure 3A). The results indicate that salusin-β-induced inhibition in sympathetic activity is independent of vagi and baroreflex and may be mediated by some central mechanisms in inhibiting the origin of sympathetic tone. Moreover, salusin-β-induced depressor and bradycardia attribute to both vagal activation and sympathetic inhibition. To confirm the roles of salusin-β in altering the sympathetic-parasympathetic balance, we further examined the effects of salusin-β in iDMV on the HRV. Bilateral microinjection of PBS into the iDMV had no significant effects on the HRV. Salusin-β increased the normalized high-frequency (nHF) but reduced the normalized low-frequency (nLF) and nLF/nHF (Figure 3B), suggesting that salusin-β in iDMV causes sympathetic inhibition and vagal activation and alters the sympathetic-parasympathetic balance.

### 3.4. Effects of Anti-Salusin-β IgG and Salusin-β Immunohistochemistry

**Figure 4:**
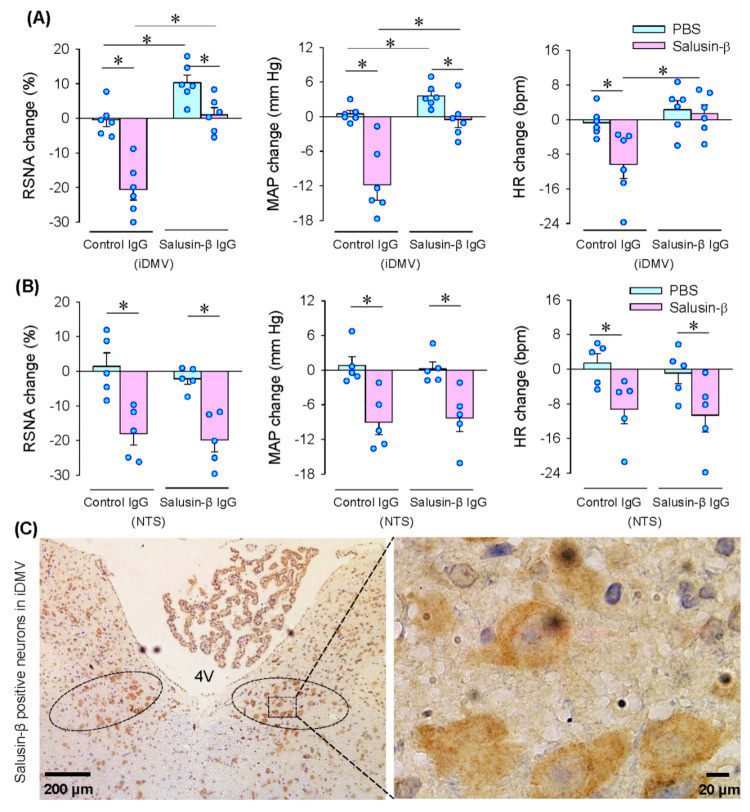
Roles of neutralization of salusin-β with antibody in iDMV or NTS and salusin-β expression in iDMV. (**A**) Effects of microinjection of control IgG (100 ng) or salusin-β IgG (100 ng) in the iDMV on the salusin-β-induced RSNA, MAP and HR changes. The pretreatment with IgG in the iDMV was carried out 10 min before microinjection of salusin-β (100 pmol) in the iDMV. (**B**) Effects of microinjection of control IgG (100 ng) or salusin-β IgG (100 ng) in the NTS on the salusin-β-induced RSNA, MAP and HR changes. The pretreatment with IgG in the NTS was carried out 10 min before microinjection of salusin-β (100 pmol) in the iDMV. (**C**) Representative images of immunohistochemistry for salusin-β (brown color) iDMV. 4V, the fourth ventricle. * *p* < 0.05.

Microinjection of anti-salusin-β IgG into the iDMV abolished the roles of the microinjection of salusin-β into the iDMV in reducing the RSNA, MAP and HR, suggesting the effectiveness of anti-salusin-β IgG in neutralization of salusin-β in the iDMV. Importantly, microinjection of anti-salusin-β IgG alone into the iDMV increased RSNA and MAP, but had no significant effect on the HR, suggesting that endogenous salusin-β in the iDMV contributes to the maintaining of the sympathetic outflow and blood pressure (Figure 4A). Furthermore, microinjection of anti-salusin-β IgG into the NTS had no significant effects on the RSNA, MAP and HR. The effects of salusin-β in the iDMV were not affected by microinjection of anti-salusin-β IgG into the NTS (Figure 4B). The results further confirmed that the effects of salusin-β on the RSNA, MAP and HR were not caused by its diffusion to the NTS. Salusin-β-positive neurons were found in the iDMV, supporting the roles of salusin-β in the iDMV (Figure 4C).

### 3.5. Reactive Oxidative Species (ROS) Production Mediates the Effects of Salusin-β in iDMV

**Figure 5:**
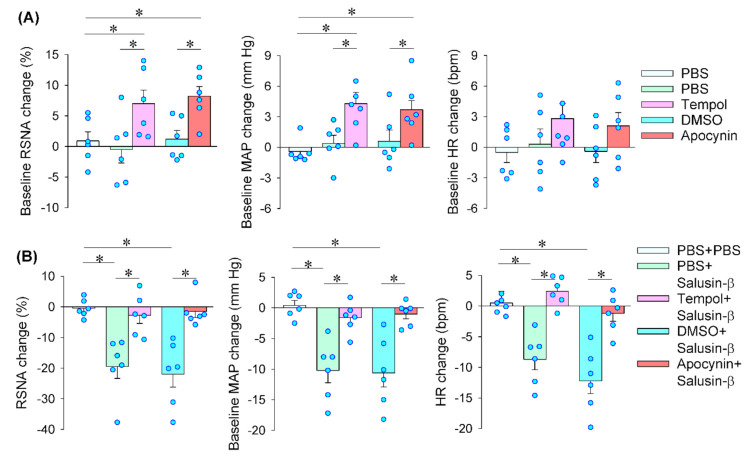
Effects of tempol and apocynin on the salusin-β-induced RSNA, MAP and HR changes. (**A**) Baseline RSNA, MAP and HR changes induced by tempol (20 nmol) and apocynin (3 nmol) in the iDMV. (**B**) Effects of pretreatment with tempol or apocynin in the iDMV on the salusin-β-induced RSNA, MAP and HR changes. The pretreatment was carried out 10 min before microinjection of salusin-β (100 pmol) in the the iDMV. * *p* < 0.05.

**Figure 6:**
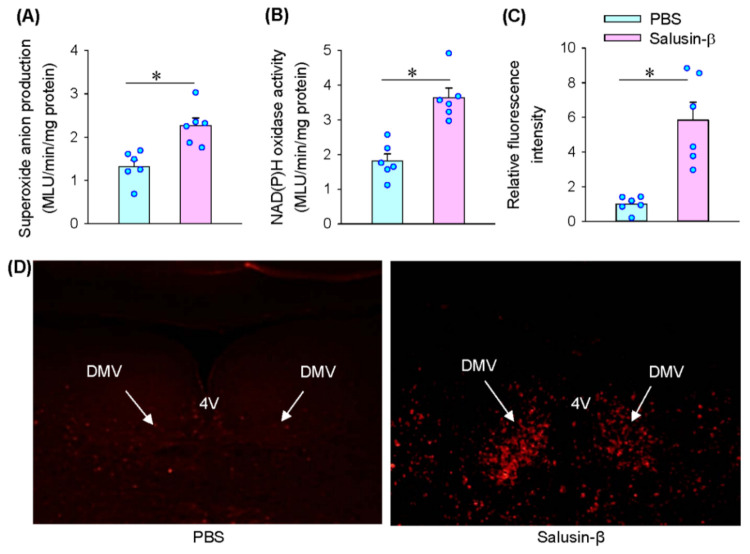
Effects of microinjection of salusin-β (100 pmol) in the iDMV on superoxide anion production and NAD(P)H oxidase activity. (**A**) Superoxide anion production. (**B**) NAD(P)H oxidase activity. (**C**) Relative fluorescence intensity according to DHE staining analysis. (**D**) Representative images showing DHE staining. 4V, the 4th ventricle. * *p* < 0.05.

Microinjection of superoxide anion scavenger tempol or NAD(P)H oxidase inhibitor apocynin into the iDMV caused increases in RSNA and MAP (Figure 5A). The results suggest that endogenous ROS production originated from NAD(P)H oxidase in the iDMV contributes to the tonic control in regulating sympathetic-parasympathetic balance. Pretreatment with tempol or apocynin into the iDMV completely abolished the roles of salusin-β in the iDMV in reducing RSNA, MAP and HR (Figure 5B). Microinjection of salusin-β in the iDMV increased superoxide anion production (Figure 6A) and NAD(P)H oxidase activity (Figure 6B). Moreover, DHE fluorescence intensity was significantly increased after the iDMV microinjection of salusin-β (Figure 6C,D). These results provided strong evidence that NAD(P)H oxidase dependent ROS production mediates the effects of salusin-β in the iDMV.

### 3.6. GABAA Receptors in PVN Mediates the Effects of Salusin-β in iDMV

**Figure 7:**
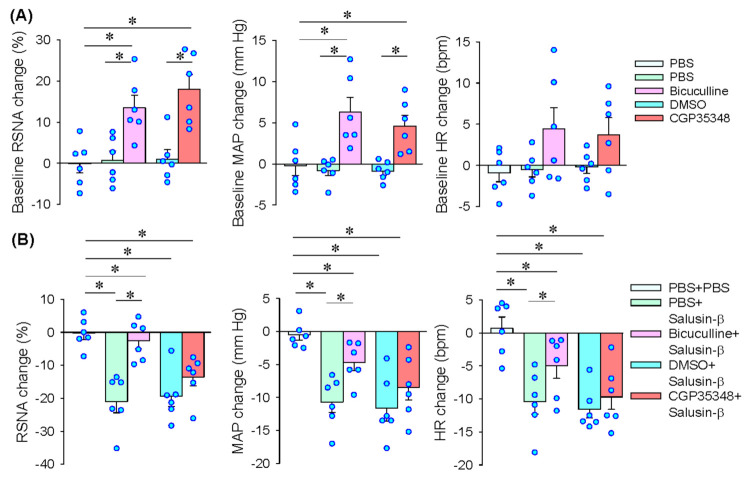
Roles of GABA receptors in the PVN in mediating the effects of salusin-β in iDMV. Microinjection of salusin-β (100 pmol) in iDMV was carried out 10 min before microinjection of bicuculline (a GABAA receptor antagonist, 100 pmol) or CGP35348 (a GABAB receptor antagonist, 10 nmol) into the PVN. (**A**) Baseline RSNA, MAP and HR changes induced by bicuculline or hydrochloride in iDMV. (**B**) Effects of pretreatment with bicuculline or CGP35348 in the PVN on the roles of salusin-β in the iDMV. * *p* < 0.05.

As mentioned above, salusin-β in the iDMV in reducing MAP and HR attributes to both vagal activation and sympathetic inhibition. However, the mechanism of salusin-β in the inhibition of sympathetic activity is unknown. It is known that the PVN is a critical integrated center in control sympathetic outflow [33]. The receptors of γ-aminobutyric acid (GABA) in the PVN is involved in inhibiting in sympathetic outflow and blood pressure [34,35]. Thus, we further investigated whether GABA receptors in the PVN might mediate the effects of salusin-β in sympathetic inhibition. Microinjection of GABA_A_ receptor antagonist bicuculline or GABA_B_ receptor antagonist CGP35348 into the PVN increased RSNA and MAP (Figure 7A), suggesting that both GABA_A_ and GABA_B_ in the PVN contribute to the tonic inhibition of sympathetic activity. Pretreatment with bicuculline almost completely abolished salusin-β-induced inhibition in the RSNA, but only attenuated salusin-β-induced depressor and bradycardia responses. However, CGP35348 had no significant effects on the roles of salusin-β in iDMV (Figure 7B). These results indicate that salusin-β-induced sympathetic inhibition is mediated by GABA_A_ receptors rather than GABA_B_ receptors in the PVN.

## 4. Discussion

The DMV is well known to be an important integrative center for the control of vagal outflow [10]. We firstly showed that salusin-β in the iDMV regulated the balance between sympathetic activity and vagal activity. Exogenous salusin-β in the iDMV reduced blood pressure and heart rate by not only the vagal activation but also the sympathetic inhibition. Neutralization of salusin-β with anti-salusin-β IgG increased sympathetic outflow and blood pressure, suggesting endogenous salusin-β in the iDMV plays a tonic role in inhibition of sympathetic activity. The direct evidence of salusin-β in inhibition of sympathetic activity was that the bilateral vagotomy only attenuated depressor and bradycardia responses of salusin-β but had no significant effect on the sympathetic activity. HRV is widely used for determine sympathetic activity and parasympathetic activity in human and animals [26,27,28]. The changes of HRV in this study provided further evidence that salusin-β in iDMV has bilateral effects on both sympathetic and parasympathetic activity. Salusin-β is widely distributed in the brain and peripheral tissues [17,36]. We found that a lot of salusin-β-positive neurons with high salusin-β expression existed in the iDMV, supporting the important role of salusin-β in the iDMV. Our previous studies have shown that salusin-β in the PVN or the RVLM increased sympathetic outflow and blood pressure in SHR but not in normal rats [22,23]. Salusin-β in NTS of SHR reduces sympathetic outflow and blood pressure in SHR [24]. It seems that the central roles of salusin-β in inhibiting or enhancing sympathetic activity are different in the different brain zones. Interestingly, salusin-β in the PVN, RVLM or NTS had no physiological roles in regulating sympathetic activity and blood pressure in normal rats, but salusin-β in the iDMV contributes to the physiological roles in regulating sympathetic and parasympathetic activity, blood pressure and heart rate in normal rats.

Salusin-β in arteries promotes ROS production, inflammation and vascular remodeling [21,37]. Excessive ROS causes several pathological processes including inflammation and cell damage [38,39,40], while an adequate amount of ROS serves as critical signaling molecules in physiological state [41]. NAD(P)H oxidase is the major ROS source in the vasculature and the PVN [29,42]. ROS in vagal neurons in DMV promotes pathogenetic processes of Parkinson’s disease [43]. However, it is still not known whether the ROS in the DMV is involved in regulating sympathetic outflow and blood pressure. Inhibiting NAD(P)H oxidase or scavenging superoxide anions in the iDMV increased RSNA and MAP, suggesting endogenous ROS production plays a tonic role in inhibiting sympathetic activity and reducing blood pressure in physiological state, which may serve as an important mechanism for prevent excessive activation in certain pathological situations. Pretreatment with NAD(P)H oxidase inhibitor or superoxide anion scavenger in the iDMV almost abolished the roles of salusin-β in reducing RSNA, MAP and HR. Furthermore, salusin-β in the iDMV increased NAD(P)H oxidase activity and increased the ROS levels in the iDMV. These results indicate that both sympathetic inhibition and vagal activation induced by the salusin-β in iDMV were mediated by the NAD(P)H oxidase-originated ROS production in the iDMV.

**Figure 8:**
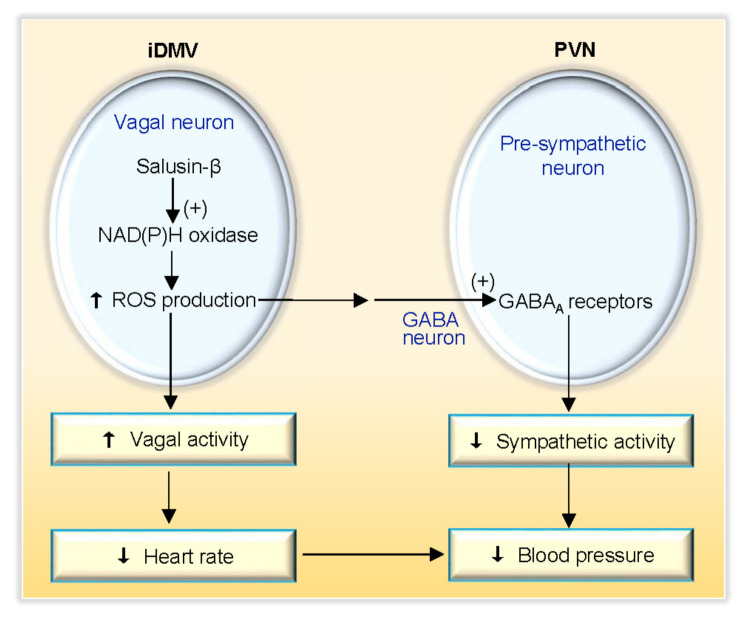
Schematic diagram showing the roles of salusin-β in iDMV and its downstream signal pathway.

It is known that there are projections between the PVN and the DMV [44]. PVN has emerged as one of the most crucial autonomic control centers in the brain [45,46]. GABA in the PVN is involved in control sympathetic activity and blood pressure [47,48]. Activation of GABA_A_ or GABA_B_ receptors in the PVN reduced sympathetic activity and blood pressure, while blockade of GABA_A_ or GABA_B_ receptors in the PVN increased sympathetic activity and blood pressure [49]. We found that blockade of GABA_A_ receptors in the PVN almost abolished the iDMV microinjection of salusin-β-induced inhibition in sympathetic activity, but only attenuated salusin-β-induced depressor and bradycardia responses. Blockade of GABA_B_ receptors in the PVN had no significant effects on the roles of salusin-β. These results indicate that salusin-β-induced inhibition in sympathetic activity is mediated by the activation of the GABA_A_ receptors in the PVN. The salusin-β-induced depressor and bradycardia responses attribute to both sympathetic inhibition and vagal activation (Figure 8). Excessive sympathetic activity plays crucial roles in the development and progress of several diseases including hypertension, chronic heart failure and chronic renal disease [50,51]. The iDMV may be involved in the excessive sympathetic activation in these diseases, which need further investigation.

In conclusion, salusin-β in the iDMV inhibits sympathetic activity and enhances vagal activity, and thereby reduces blood pressure and heart rate. The effect of salusin-β on vagal activation is mediated by NAD(P)H oxidase activation-dependent superoxide anion production in the iDMV. The effect of salusin-β on sympathetic inhibition is mediated by NAD(P)H oxidase-originated superoxide anion production in the iDMV and the following GABA_A_ receptor activation in the PVN. Endogenous salusin-β and ROS production in the iDMV play a tonic role in inhibiting sympathetic activity. The findings reveal the novel mechanism for the central regulation of sympathetic-parasympathetic balance.

## Data Availability

The data presented in this study are available on request from the corresponding author on reasonable request.
